# Optic Disc Edema and Elevated Intracranial Pressure (ICP): A Comprehensive Review of Papilledema

**DOI:** 10.7759/cureus.24915

**Published:** 2022-05-11

**Authors:** Louis Reier, James B Fowler, Mohammad Arshad, Hamid Hadi, Eric Whitney, Anthony V Farmah, Javed Siddiqi

**Affiliations:** 1 Neurosurgery, Desert Regional Medical Center, Palm Springs, USA; 2 Neurosurgery, Riverside University Health System Medical Center, Moreno Valley, USA

**Keywords:** benign intracranial hypertension, pseudotumor cerebri syndrome (ptcs), intracranial idiopathic hypertension, optic disc swelling, papilledema

## Abstract

Papilledema is a swelling of the optic disc secondary to elevated intracranial pressure (ICP). We analyzed 79 peer-review journal articles and provided a concise summary of the etiology, epidemiology, pathophysiology, clinical presentation, evaluation, natural history, differential diagnosis, treatment, and prognosis of papilledema. Only studies written in English with the full text available were included. Although many etiologies of papilledema exist, idiopathic intracranial hypertension is the most common and, thus, a large focus of this review.

## Introduction and background

Papilledema refers to optic disc swelling secondary to elevated intracranial pressure (ICP) [1]. Optic disc edema occurs as a result of reduced perfusion to retinal ganglion axons and subsequent swelling of the axons with leakage of cellular contents into the extracellular space of the optic disc [2,3]. Typical symptoms of papilledema are attributable to elevations in ICP, and appropriate workup is directed at identifying the underlying etiology [1,4,5]. While numerous etiologies have been associated with papilledema, it is mostly defined in the setting of idiopathic intracranial hypertension (IIH). The natural history, treatment, and prognosis varies depending on the underlying etiology. Regardless of the cause, papilledema should be treated promptly to avoid permanent injury.

## Review

Etiology

The etiologies of papilledema are vast since, technically, almost every disease that elevates intracranial pressure (ICP) can cause papilledema [1,6]. Possible causes include an increase in cerebrospinal fluid (CSF) production, decrease in CSF resorption, obstruction of CSF flow, central nervous system (CNS) space-occupying lesion (e.g., brain tumor or hematoma), diffuse cerebral edema, malignant hypertension, pharmacologic effects, and IIH [1,7,8].

The most common etiology is IIH [6]. A retrospective study done by Crum et al. evaluated the etiology of papilledema in patients presenting to an optometrist and ophthalmologist clinic over a 24-year period. After excluding patients who had a previous diagnosis that would explain fundoscopic findings of papilledema, they found 87% of cases to be from IIH and 13% from a secondary cause of elevated ICP [6].

Epidemiology

The epidemiology of papilledema varies based on the underlying etiology [9]. In general, papilledema is seen across all ages, races, ethnic groups, as well as both genders [1]. Most epidemiological studies were done on papilledema focusing specifically on IIH. IIH is unique and differs from other causes of papilledema as there is no identifiable cause of elevated ICP.

When papilledema is secondary to IIH, 90% affected are females, the average age of diagnosis is 29, and the average BMI is 39.9 [4]. Additionally, 5% have a family member with the disease, suggesting the possibility of genetic contribution [4]. Caucasians are the most common race affected (accounting for 65% of those diagnosed), followed by African Americans [4]. The country with the lowest incidence reported is Japan, suggesting the disease occurs less frequently among Asian populations [10].

The incidence of IIH in USA differs slightly from that of other countries, which is thought to be due to obesity. The estimated incidence within different demographics in USA is listed in Table 1 [1,10].

**Table 1:** Incidence of idiopathic intracranial hypertension (IIH) in USA The information used in this table was obtained from the following sources: [1,10]

Demographics	Incidence
Among the general population	0.9 per 100,000
Women of childbearing age	3.5 per 100,000
Obese women of childbearing age, with an ideal body weight of 10-20%	13 per 100,000
Obese women of childbearing age, with an ideal body weight >20%	19.3 per 100,000

Table 2 is a summary of the incidence of IIH among various countries. We provide the incidence of IIH among both the general population as well as in women. We organized this table by year of study.

**Table 2:** Incidence of idiopathic intracranial hypertension (IIH) among various countries

Estimated overall incidence among population	Estimated incidence in women	Country	Year
1.7 per 100,000 [11]	3.6 per 100,000 (women of all ages)	Libya	1984
2.2 per 100,000 [12]	12 per 100,000 (women of childbearing age)	Libya	1993
0.9 per 100,000 [12]	3.5 per 100,000 (women of childbearing age)	USA	1993
0.9 per 100,000 [13]	13 per 100,000 (women of childbearing age)	USA	1998
0.3 per 100,000 [14]	Not studied	Japan	2000
0.50 per 100,000 [15]	1.56-1.98 per 100,000 (women of childbearing ages)	UK	2001
0.94 per 100,000 [16]	4.02 per 100,000 (women of childbearing age)	Israel	2001
2.3 per 100,000 [17]	4 per 100,000 (women of all ages)	Wales	2003
0.28 per 100,000 [18]	0.65 per 100,000 (women of childbearing age)	Italy	2004
1.56 per 100,000 [19]	2.86 per 100,000 (women of all ages) 11.9 per 100,000 (obese women of childbearing age)	UK	2011
2.18 per 100,000 [20]	4.14 per 100,000 (women of childbearing age)	Oman	2011
7.8 per 100,000 [17]	12-14 per 100,000 (women of all ages)	Wales	2017

Pathophysiology

The optic nerve is composed of approximately 770,000 to 1.7 million retinal ganglion cell axons and glial cells that serve to transduce information from the retinal photoreceptors to nine primary visual nuclei in the brain as part of the visual field pathway [21]. The optic nerve is encompassed by all three meningeal layers and, therefore, continuous with the subarachnoid space of the brain rather than peripheral epineurium, perineurium, and endoneurium, making it a component of the central nervous system and therefore with limited regenerative capability [22].

While initial theories proposed that high ICP resulted in compression of the subarachnoid portion of the central retinal vein, leading to optic disc edema via venous obstruction (termed mechanical theory), the theory was challenged with the advent of electron microscopy (EM) [23]. EM demonstrated the optic disc edema of papilledema was primarily intra-axonal, affecting axoplasmic energy-dependent axoplasmic transport [24]. Stasis of intra-axonal fluid results in swelling of the axons and leakage of cellular contents into the extracellular space of the optic disc, giving rise to optic disc edema. Reduced perfusion of axons results in a secondary phenomenon of venous obstruction and dilation, nerve ischemia, and vascular telangiectasias [2,3].

The doctrine of venous hypertension gave way to axoplasmic stasis, but controversy still exists regarding the underlying mechanism with proponents that favor direct compression by elevated ICP (mechanical theory) verse reduced perfusion of axons (ischemic theory) [25].

Clinical presentation 

Nonspecific Etiology

Since papilledema presents in the setting of elevated ICP, regardless of the underlying etiology, typical complaints include headache, nausea, vomiting, and an array of visual symptoms [1,4,26,27]. When a secondary source of elevated ICP is the cause, patients typically have identifiable image findings and/or focal neurological deficit(s) that localize to the offending anatomical region of interest [6]. If symptoms cannot be explained by another diagnosis, image findings are unremarkable, and no neurological deficits are appreciated on examination, then IIH should be considered and worked up appropriately [5].

Secondary to IIH

Headache is the most common complaint in patients with IIH [4,5,28]. In the Idiopathic Intracranial Hypertension Treatment Trial, 84% of patients complained of a headache [4]. Similarly, in an article by Puffer et al., 90% of patients with IIH presented with a headache [28]. Even though headache characteristics are variable, 68% describe features similar to that of a migraine [5]. Other common headache characteristics are bilateral involvement, more common in the mornings, positional (worse when lying down, better when upright), and intensify with coughing or straining [5,9]. The frequency of exacerbation and severity typically progresses over time [4,5,28].

Transient visual obscurations are the second most common complaint, occurring in 68-72% of patients, with episodes lasting seconds and frequently occurring daily [4,5,29]. Pulsatile tinnitus is the third most common reported symptom, occurring in 52-61% of patients [5]. Other reported symptoms, from increasing to decreasing frequency, include: back pain (53%), dizziness (52%), photophobia (48%), neck pain (42%), vision loss (32%), nocturia (30%), cognitive disturbance (20%), radicular pain (19%), and diplopia (typically horizontal) (18%) [4].

There are two important findings unique to IIH which clinically distinguish it from other causes of papilledema. First, no localizing deficits will be appreciated on examination (apart from cranial nerve six palsy, which has an estimated prevalence of 12% among adult patients) [5,30]. Second, all imaging of the head and neck will all be negative.

The most widely known diagnostic criteria is the Modified Dandy (Table 3) [31-33].

**Table 3:** Modified Dandy criteria for diagnosis of idiopathic intracranial hypertension (IIH) The information used in this table was obtained from the following sources: [31,33] CTV: computerized tomographic venography; ICP: intracranial pressure; MRV: magnetic resonance venography; CSF: cerebrospinal fluid

Modified Dandy criteria
Signs and symptoms of elevated ICP (e.g., headache, nausea, vomiting, transient visual obscurations, and papilledema on fundoscopic exam).
Patient is alert and oriented with no signs of altered mental status. No neurological deficits on exam, with the exception of abducens nerve palsy (can be unilateral or bilateral).
Elevated ICP based on lumbar puncture opening pressure >25 cmH20. CSF cytology normal.
Neuroimaging unremarkable for identifying a culprit of increased ICP. CT head negative for hydrocephalus, hemorrhage, or hematoma. MRI/MRV head and neck negative for underlying mass, structural or vascular lesion. CTV may be used in place of MRV.
No other explanation for elevated ICP.

Fundoscopic Evaluation and Expected Findings

Identifying papilledema on fundoscopic examination is needed to make the diagnosis [34]. Optic disc findings provide information about the severity and chronicity of papilledema. Additionally, it provides guidance in clinical decision-making and disease management [4,5,28,34]. The most common grading scale was published in 1982 by Frisén [35]. This scale ranges between the numeric values of zero to five, with zero representing normal optic disc and five being the most severe form of papilledema [35].

**Figure 1:**
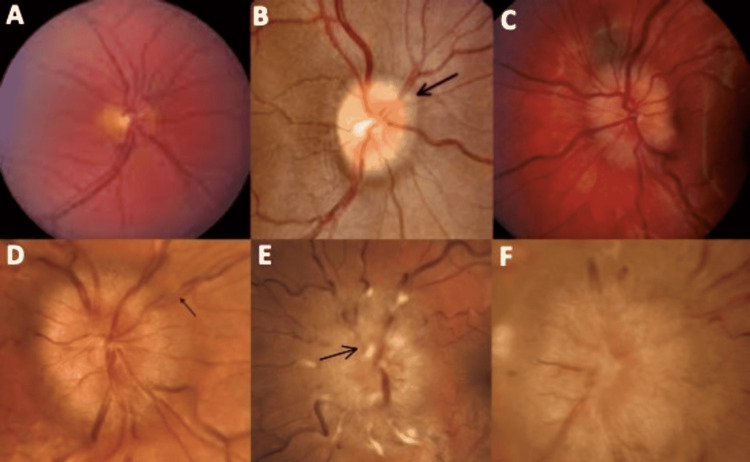
Stages of papilledema on fundoscopic exam (A) Frisén grade zero: normal optic disc. (B) Frisén grade one: the amount of edema is minimal. "C" shaped greyish halo surrounding the disc (arrow) with spared temporal disc margin. (C) Frisén grade two: the amount of edema is marginal. Halo is now circumferential. Elevation of nasal border. (D) Frisén grade three: the amount of edema is moderate. Circumferential halo. All borders become elevated (not including the cup). One or more segment(s) of blood vessels leaving the disc becomes obscured (arrow). (E) Frisén grade four: the amount of edema becomes evident. Circumferential halo. All borders elevated (including the cup). One or more major vessels on the disc becomes obscured (arrow). (F) Frisén grade five: all of grade 4 features plus partial or total obscuration of all vessels on and leaving the disc. The photographs used for this figure were obtained from the following source: [36] The information obtained for the descriptive portion of this figure was obtained from the following source: [35]

Figure 1 is a modification of the Frisén grading scale that we made to include the most prominent findings of each stage [35]. Both descriptive and ophthalmoscopic findings are included [36].

Regardless of the Frisén grade or severity of symptoms, when papilledema is detected, it is recognized as a medical emergency since the progression of the disease can lead to irreversible vision loss [29]. An immediate multidisciplinary evaluation should be provided promptly [34]. 

Evaluation

The evaluation of papilledema focuses on identifying the underlying etiology and ruling out pseudopapilledema. After the identification of edematous optic discs, neuroimaging is often employed. MRI and magnetic resonance venography (MRV) of the brain are often used to evaluate for any mass lesion, cerebral sinus thrombosis, or surrogate evidence of increased ICP. MRI is preferred over CT as MRI provides superior detail while minimizing the patients' radiation exposure. MRI findings suggestive of elevated ICP include the following: posterior globe flattening, distention of the perioptic CSF space, empty or partially empty sella, and transverse venous sinus thrombosis [34,37].

When there are no contraindications, lumbar puncture with opening pressure should be performed [34,38]. CSF analysis can help rule out neoplastic or infectious etiologies, and lumbar puncture may provide transient relief of headaches [34]. CSF pressure above 25 cm H_2_O is concerning for increased ICP [34].

Visual field testing with perimetry can be helpful in detecting subtle visual field abnormalities, monitoring sequala, and evaluating response. The size of the blind spot is an indirect measure of disc edema [39].

Fluorescein angiography (FFA) relies on fluorescence to evaluate the vasculature of the retina. FFA can visualize dynamic effects of vascular filling as well as blood vessel leakage due to endothelial damage, inflammation, and neovascularization or raised ICP [40]. Mild to moderate degrees of papilledema must be present to detect changes in FFA. The first change often involves the blurring of the disc margin, usually beginning inferiorly. Later moderate or severe changes include masking of the choroidal fluorescence in the peri-papillary zone and the presence of deep optic disc fluorescence [41]. Chang et al. concluded that FFA is the most accurate modality for differentiating optic disc edema from pseudopapilledema, with a sensitivity between 83-97%, but did stress the limitations of clinical interpretation [42].

Quantitative pupillometry or neurologic pupil index (NPi) quantifies the reactivity of the pupillary response. This non-invasive method has been shown to correlate with elevated ICP in both instances of known pathology (i.e., trauma) and with IIH [43,44]. Although there are no current studies in the literature confirming a correlation between papilledema and NPi, quantitative pupillometry may be used as a surrogate to evaluate for papilledema [45].

Orbital ultrasound provides a rapid, minimally invasive modality-assessing papilledema, correlating optic nerve sheath width and ICP. Nerve sheath width >3.3mm in an adult patient was considered positive, with a sensitivity of 90% and specificity of 79% for detecting pseudopapilledema [46].

Optical coherence tomography (OCT) is a potential tool to quantify changes in papilledema and monitor treatment interventions. The imaging technique quantifies nerve fiber layer and total retinal thickness and has been used as an adjunct modality to differentiate pseudopapilledema from papilledema [47].

Natural history

The natural history of papilledema is variable and depends on the underlying condition leading to ICP elevation. Specific causes of papilledema are discussed in the “etiology” and “differential diagnosis” sections. In general, optic disc edema secondary to elevated ICP occurs no earlier than 24 hours and usually by seven days [48]. Following ICP reduction, well-developed papilledema typically resolves in 1.5 to 2.5 months [49]. If the underlying disease is not addressed and ICP remains elevated, optic nerve atrophy leading to permanent vision loss may ensue [50]. Once papilledema has progressed to a chronic state, the ability to recover visual function drastically decreases [51].

There are a limited number of studies in the literature on the natural history of papilledema. We found most publications focused specifically on IIH, cerebral venous sinus thrombosis (CVST), and fundoscopy findings.

Natural History of Papilledema Secondary to IIH

Papilledema in the setting of IIH usually resolves after a few months to a few years post treatment, while persisting in roughly 15% and reoccurring in roughly 10% of patients [52]. Eighty percent of patients report improvement in headaches following CSF diversion surgery [53]. With treatment, visual deficits usually resolve. If left untreated, permanent vision loss occurs in approximately 25-50% of people [54].

Natural History of Papilledema Secondary to Venous Sinus Thrombosis

Liu et al. did a multicenter retrospective cohort on the natural history of papilledema and vision outcomes in the setting of cerebral venous sinus thrombosis (CVST). Results of their study showed the following: in the setting of CVST, papilledema can persist for a long duration [48]. The grade of papilledema on initial presentation is often severe (average Frisén score of 2.7) [48]. Progression of papilledema occurred in 21.5% of patients over an average span of 55.3 days (range: 6-180 days) [48]. Progression of papilledema and Frisén grade ≥3 were both associated with a higher risk of permanent visual field loss [48]. When papilledema resolved, it typically took just over six months (range: 21-551 days), and upon resolution, 40% of patients still had some form of deficit seen on formal visual field testing [48].

Natural History of Fundoscopy Findings in Papilledema

Early papilledema clinically has persevered color and visual acuity with early disc swelling, an absence of venous pulsations, and early opacification of the nerve fiber layer [50].

Fully developed papilledema can lead to visual field cuts, pulsatile tinnitus, and decreased vision secondary to fluid in the macula. On fundoscopy, there is an elevated disc surface, choroidal folds, exudates, and hemorrhage [50].

In chronic papilledema, hemorrhages resolve, disc hyperemia becomes grey, small exudates appear on the disc surface, optociliary vessels develop, and further visual impairment is seen [50].

Differential diagnosis

Optic disc edema refers to swelling of the nerve fiber layer at the optic nerve head and may be due to numerous etiologies. This is clinically distinct from true papilledema, which should be reserved for patients with elevated optic nerve head because of increased ICP. Conditions associated with papilledema are outlined in the "etiology" section.

Numerous conditions have been associated with optic disc edema without elevation in ICP. This can be seen with inflammatory, infiltrative, infective, or compressive pathologies among others. Listed in Table 4 are some cited etiologies found in the literature. 

**Table 4:** Conditions that can cause optic disc edema in the setting of normal intracranial pressure (ICP)

Conditions causing optic disc edema without elevated intracranial pressure
Hyperviscosity, hypotension, and blood loss [55]
Toxic optic neuropathies producing disc edema early in their course have been described with methanol, ethambutol, ethylene glycol, and other toxins [56]
Optic nerve compression by infiltrated extraocular muscles in severe thyroid ophthalmopathy [57]
Cerebral venous sinus thrombosis (CVST) [58]
Anterior ischemic optic neuropathy [59]
Optic neuritis of the anterior portion of the optic nerve, from demyelinating diseases. Neuroretinitis, inflammation of the retina with macular exudates, from viral syndromes, toxoplasmosis, sarcoidosis, systemic lupus erythematosus among others [60-62]
Central retinal vein occlusion [63]
Malignancy, including meningioma, glioma, hamartoma, dermoid, lymphoma and leukemia [64,65]
Leber hereditary optic neuropathy [66]
Prior eye surgery [67]
Prior radiation [68]

Pseudopapilledema is defined by an elevation of the nerve head without edema of the nerve fiber layer and portends drastically different clinical consequences than true papilledema. The optic nerve may be elevated because the nerve enters the eye at an anomalous oblique angle, usually elevating the nasal aspect. Hyperopia is a common type of refractive error characterized by a globe with a shorter axial length and often with a small optic cup, which may have the appearance of an elevated nerve head due to crowding of the nerve fibers [69]. The nerve fiber layer, which is normally translucent, may be partially myelinated from anomalous migration of oligodendrocytes beyond the lamina cribosa, blurring the margins of the optic disc [69]. Other causes of elevated optic disc head include peripapillary astrocytic hamartomas and other peripapillary tumors [70,71]. One of the most common causes of pseudopapilledema is optic disc drusen: conglomerates of mucopolysaccharides and proteinaceous material that develop within nerve tissue and may lead to an elevated disc head. The accumulation of extracellular deposits is thought to be related to altered axoplasmic flow and is often associated with advanced age (seen in 2% of the population) or inherited conditions [72]. Unlike papilledema, pseudopapilledema typically has a benign clinical course, lacks visual symptoms, and no treatment is usually required [73].

Treatment

Treatment of papilledema is based on identifying and addressing the underlying cause [9]. For example, presence of a space occupying mass may indicate surgical resection [9]. Venous sinus thrombosis is typically treated with anticoagulation and/or endovascular stenting [9,27,74-76]. If the workup for a secondary cause of elevated ICP is unremarkable and IIH is diagnosed, the severity of the patient's symptoms guides management [4,9].

IIH: Nonsurgical Treatment Options

If visual changes are mild, first-line treatment is weight loss via diet and exercise, in combination with acetazolamide [4,9]. Weight loss is the only disease-modifying therapy to date [26]. A reduction of 5-10% of total body weight is associated with improvement in signs and symptoms [1]. When combined with weight loss, acetazolamide is the only medication that has been shown in a controlled trial to benefit patients experiencing visual changes [1,4]. Topiramate is used for symptomatic control of headaches; however, some studies have shown it also has the added benefit of weight loss [1]. Corticosteroids and serial lumbar punctures (or lumbar drain) can be used as a temporizing means in those with a fulminant presentation [51,77].

IIH: Surgical Treatment Options

If refractory to conservative therapy and visual symptoms progress (or if ever fulminant), surgical intervention is indicated [4,9]. Surgical options include optic nerve sheath fenestration (ONSF), permanent CSF diversion shunting procedures such as ventriculoperitoneal shunt (VPS), or lumbar peritoneal shunt (LPS), and endovascular venous sinus stenting (EVSS) [4,9,51]. 

ONSF: This surgery involves directly decompressing the optic nerve, thus is an effective option demonstrating superior outcomes for those with predominantly visual symptoms in the acute setting [51]. Once papilledema has progressed to a chronic state, the ability to recover visual function drastically decreases, and ONSF becomes a less effective option [51]. In comparison to CSF diversion procedures, it is inferior at controlling headaches [51].

CSF diversion (VPS and LPS): Both are long-term treatment options with outcomes similar to ONSF. The difference is that CSF diversion surgery results in better outcomes compared to ONSF when symptoms are predominantly headaches, whereas ONSF has better outcomes when complaints are predominantly visual [51].

EVSS: This may be done for cases in which stenosis of the dominant or codominant transverse sinus is identified [75]. Transverse sinus stenosis is often seen in patients with IIH. Whether transverse sinus stenosis is the underlying cause or develops secondarily to IIH is currently debated. Regardless of the pathophysiology, when stenosis is present with a pressure gradient ≥9 mmHg, EVSS has shown excellent results in symptomatic improvement among those who have failed conservative therapy [75]. 

Prognosis

The prognosis of papilledema is dependent on the underlying cause. However, when papilledema is caused by IIH, vision loss is the most feared outcome and the primary variable analyzed when determining patient prognosis [78]. It is important to understand which factors place patients at a higher risk of vision loss. Not only is this important for prognostic purposes, but also for physicians to provide optimal patient care. 

A study done by Corbett et al. analyzed vision loss in patients with IIH to determine which factors placed patients at greatest risk [77]. They followed 57 patients with a known diagnosis of IIH for five to 41 years. At the conclusion of their study, 14 of the 57 patients developed either complete vision loss or "severe visual impairment" [78]. Seven of those 14 patients did not develop their visual deficit until months to years after they were first diagnosed, suggesting vision loss can occur at any stage of the disease [78]. Most importantly, 13 of 57 patients studied had a past medical history of hypertension, and eight out of these 13 patients became blind [78]. This shows that one of the greatest risk factors for having a poor prognosis among IIH patients is hypertension [78].

Other key elements that contribute to a patient’s prognosis include the following: acuity of symptom onset and symptom progression (rapid symptom onset and progression can lead to poor prognosis if immediate intervention is not provided); the amount of vision loss on presentation (the more prominent presenting vision loss is, and the rate at which vision deteriorates correlates with a worse prognosis); and Frisén grade at presentation (higher Frisén grade correlates with a worse prognosis) [79].

## Conclusions

Papilledema is swelling of the optic disc secondary to elevated ICP. Papilledema is a medical emergency, as permanent vision loss can ensue without immediate intervention. It is seen across all ages, races, ethnic groups, and affects both genders. IIH is the most common cause of papilledema and is usually seen in obese Caucasian women of childbearing age. The most common signs and symptoms patients present with include headaches, nausea, vomiting, and an array of visual complaints. IIH is a diagnosis of exclusion based on the modified Dandy criteria. A fundoscopic exam is essential to making the diagnosis and can provide information about the acuity and severity of papilledema, as well as guidance in clinical management. Treatment of papilledema is based on identifying and addressing the underlying cause. If IIH is deemed the cause, non-surgical options include weight loss, diet, and exercise in combination with acetazolamide. Surgical options include ONSF, EVSS, and CSF diversion. When signs and symptoms are predominantly visual, ONSF is associated with better outcomes. However, when headaches are the primary complaint, CSF diversion is superior. EVSS has shown promising results for treating transverse sinus stenosis when a pressure gradient is present. The prognosis of papilledema varies and depends on the acuity of symptom onset, presenting Frisén grade, amount of vision loss at presentation, and degree of symptom progression.
